# Ida-1, the *Caenorhabditis elegans* Orthologue of Mammalian Diabetes Autoantigen IA-2, Potentially Acts as a Common Modulator between Parkinson’s Disease and Diabetes: Role of Daf-2/Daf-16 Insulin Like Signalling Pathway

**DOI:** 10.1371/journal.pone.0113986

**Published:** 2014-12-03

**Authors:** Soobiya Fatima, Rizwanul Haque, Pooja Jadiya, Shamsuzzama, Lalit Kumar, Aamir Nazir

**Affiliations:** Laboratory of Functional Genomics and Molecular Toxicology, Division of Toxicology, CSIR-Central Drug Research Institute, Lucknow, Uttar Pradesh, India; Johns Hopkins University, United States of America

## Abstract

The lack of cure to age associated Parkinson’s disease (PD) has been challenging the efforts of researchers as well as health care providers. Recent evidences suggest that diabetic patients tend to show a higher future risk for PD advocating a strong correlation between PD and Diabetes, thus making it intriguing to decipher common genetic cues behind these ailments. We carried out studies on *ida-1*, the *C. elegans* orthologue of mammalian type-1 diabetes auto-antigen IA-2 towards achieving its functional workup vis-à-vis various associated endpoints of PD and Diabetes. Employing transgenic *C. elegans* strain expressing “human” alpha synuclein (NL5901) under normal and increased glucose concentrations, we studied aggregation of alpha synuclein, content of dopamine, expression of dopamine transporter, content of reactive oxygen species, locomotor activity, nuclear translocation of FOXO transcription factor Daf-16, and quantification of Daf2/Daf-16 mRNA. Our findings indicate that *ida-1* affords protection in the studied disease conditions as absence of *ida-1* resulted in higher alpha-synuclein aggregation under conditions that mimic the blood glucose levels of diabetic patients. We also observed reduced dopamine content, decreased motility, defective Daf-16 translocation and reduced expression of Daf-2 and Daf-16. Our studies establish important function of *ida-1* as a modulator in Daf-2/Daf-16 insulin like signalling pathway thus possibly being a common link between PD and Diabetes.

## Introduction

Age associated neurodegenerative diseases (NDs) lack a complete cure hence posing huge challenge to researchers and health care providers alike. Parkinson’s disease (PD), one of the most common condition amongst NDs, affects neurons within substantia nigra leading to multiple debilitating health events. Drawing the complexities further, are the number of experimental and epidemiological findings that present evidences on association of PD with diabetes – another such ailment that poses immense health burden particularly in elderly population [1]. Research observations are increasingly making us believe that multiple events of diabetes including mitochondrial dysfunction, metabolic inflammation and altered insulin signalling cause neuronal degeneration in diabetic subjects [2]. The detailed understanding of molecular mechanisms behind such association, however, is yet to be achieved.

Functional genomics approaches employing model system *Caenorhabditis elegans* (*C. elegans*) provide a facile platform for understanding mechanistic cues behind such important biological processes [3]. Transgenic *C. elegans* strains, particularly the one expressing ‘human’ alpha synuclein (α-syn) tagged to fluorescent reporter gene, have been shown to exhibit molecular events similar to that of PD patients; the strain exhibits aggregation of α-syn, dopamine deficit and elevated oxidative stress [4]. Researchers have also created models that mimic the events of diabetes; strains fed with specific concentrations of glucose have been shown to exhibit phenotypes relevant for studying genetic events of the ailment [5]. Further, the appreciable orthology of genes between *C. elegans* and humans, make this model precious for carrying out studies on the genetic mechanism and association of PD with diabetes [3].

In the present study, we chose to explore the *C. elegans* orthologue of mammalian genes *IA-2* and phogrin- *ida-1* (Islet cell Diabetes Autoantigen), which encodes protein tyrosine phosphatase like receptor spanning the membrane of dense core vesicles [6]. These receptors are homologous to mammalian *IA-2* (Insulinoma Associated protein-2)/*ICA-512*, and *IA-2β* (phogrin) that act as type1 diabetes (insulin dependent diabetes) auto antigen, i.e. they are considered as markers of the disease and auto antibodies are expressed prior to the appearance of clinical symptoms of the disease [7], [8]. However, the role of IA-2 and IA-2β in the pathogenesis of insulin dependent diabetes mellitus is not positively correlated [9]. The components IA-2 and IA-2β are trans-membrane protein-tyrosine phosphatases (PTP’s), but differ from typical PTP’s in a manner that these membrane proteins lack phosphatase activity because of amino acid substitution in the catalytic domain and these substitutions are evolutionary conserved [10]. The domain structure of IA-2 family proteins is highly conserved in species like humans, zebra fish, drosophila, and *C. elegans* [9], [11] **.** Thus, the studies performed on *ida-1* in *C. elegans* provide an initial framework of understanding possible link between PD and diabetes; and its implicated role in humans. Ida-1 is reported to be involved in acetylcholine release and Insulin Like Signalling (ILS) [10], [12] **.** Recent work suggests that Ida-1 has role in regulation of release of dense core vesicles (DCV), since *IA-2* interacts genetically with *UNC-31/CAPS* and in the enhancement of the weak alleles involved in ILS pathway [10]. The interaction with Calcium Activated Protein for Secretion (CAPS) indicates a possible role of Ida-1 in DCV pathway either at a level of hormone processing or maturation; or hormone sorting and loading to DCVs; or DCV trafficking and exocytosis [9] **,** providing cues for its mechanistic approach towards neurotransmission and glucose metabolism. *ida-1* has been reported to interact genetically with four genes viz *unc-31*/CAPS, *unc-64*/syntaxin, insulin like receptor *daf-2* and insulin like ligand *daf-28*. Insulin signalling pathway/daf signalling pathway in *C. elegans* has been extensively studied and is thought to be a central determinant of life span since many other pathways either depend or converge on insulin/IGF pathway transcription factor DAF-16/FOXO [13]. Ida-1 has important role in insulin/IGF pathway since silencing of *ida-1* has been shown to reduce the expression of DAF-16 which in turn is indicative of reduced chaperone activity that mediates assisting of properly folded proteins [14].

Hence the present study aims at carrying out a detailed work-up on *ida-1*, employing model system *C. elegans*. The phenotypic endpoints associated with PD were studied under various conditions towards exploring the mechanistic involvement of DAF-2/DAF-16 ILS pathway in the observed functions of Ida-1.

## Materials and Methods

### 1. *C. elegans* culture and maintenance

Standard conditions were followed for *C. elegans* propagation as described [15]. Briefly, worms were grown on a diet of *Escherichia coli* OP50 seeded Nutrient Growth Medium (NGM), which was prepared by adding 3 g of sodium chloride, 2.5 g of peptone and 17 g of agar to 975 mL double distilled water which was then autoclaved for about 40–50 minutes at 15 psi. The media was then allowed to cool to 55–60°C and then under sterile conditions, 1 mL of cholesterol (5 mg/ml of absolute alcohol); 1 mL of 1M calcium chloride; 1 mL of 1M magnesium sulphate; and 25 mL of 1M potassium dihydrogen phosphate (pH adjusted to 6) was added to it. The reagents were mixed well enough and the media was then used for culturing of the nematodes. In this study, wild type bristol N2 and other strains viz. NL5901 (transgenic strain expressing YFP in muscles with human α-syn {unc-54:: alpha synuclein::YFP+unc-119}); TJ356 (transgenic strain expressing GFP with DAF-16{integrated DAF-16:: GFP roller strain}); BZ555 (transgenic strain expressing GFP with *dat-1* {P*dat-1*::GFP}); VC226 (*ida-1* knockout strain{*ida-1*(ok409) knockout}); were used and propagated at 22°C. These strains were obtained from the *Caenorhabditis* Genetics Center (University of Minnesota, MN, USA).

### 2. Embryo isolation for obtaining synchronous nematode population

Alkaline sodium hypochlorite method was employed to isolate synchronized population of various strains of *C. elegans*. In this method, worms after being washed with M-9 buffer were treated with axenizing solution (2 mL of sodium hypochlorite and 5 mL of 1M sodium hydroxide solution) which dissolves the worm bodies to expose embryos in live condition, which were then washed twice in M9 buffer and processed further as required [16].

### 3. RNAi induced gene silencing

RNAi induced silencing of gene *ida-1*, was carried out by raising the worms on *Escherichia coli* expressing the dsRNA corresponding to *ida-1* [17], [18]. *ida-1* RNAi clones were obtained from RNAi Ahringer library purchased from SA Biosciences, Cambridge, UK. Briefly, bacterial clone expressing dsRNA targeted towards *ida-1* was cultured overnight in LB media containing 50 µg/mL ampicillin (Sigma, Cat. no. A0166) and then seeded directly on NGM plates containing 1 mM IPTG (Fermentas, Cat. no. R0393) and 25 mg/L carbenicillin (Sigma, Cat. no. C1389) and incubated overnight at 37°C. The synchronously obtained embryos were placed onto these plates for silencing of *ida-1*.

### 4. Treatment of worms with high and low glucose concentrations

High glucose concentration was created by addition of dextrose (Merck, Cat. no. 346351) in the medium on which worms were raised to mimic the glucose concentration level of 14 mM/L as in the diabetic patients and glucose concentration was restricted by use of 2-deoxy-D-glucose (DOG) (Sigma, Cat. no. D1379) [5], [19]. Briefly, 0.072 g of dextrose was weighed and dissolved in 1 mL sterile water. From this stock solution, 150 µL was dissolved in 1L NGM so as to obtain the 14 mM/L glucose concentration. For limiting glucose availability 50 mM concentration of DOG was selected, as observed in gradient based study previously (data not reported). Briefly, 16 mg of DOG was weighed and dissolved in 200 µL distilled water to make a stock concentration of 500 mM and then diluted upto 50 mM and seeded along with the bacterial culture onto the NGM plates. Worms were then allowed to grow on these plates at 22°C incubation for 48 h.

### 5. Analysis of α-syn aggregation

α-syn aggregation was observed in the transgenic strain NL5901 for the *ida-1* knockdown, high glucose and low glucose treated conditions, and compared to the control worms raised on *Escherichia coli* OP50 [20]. Well-fed worms obtained after 48 h incubation were washed with M-9 buffer. The imaging for α-syn aggregation was carried out by fluorescent microscope (Carl Zeiss, AxioVision M2) after immobilizing the worms with 100 mM sodium azide (Sigma, Cat no. 71289). The fluorescence intensity of the images was quantified by Image J software (Image J, National Institutes of Health, Bethesda, MD)**.**

### 6. Estimation of dopamine content using nonanol repulsion assay

The chemotactic activity of the nematode was estimated by nonanol repulsion assay which provides with a quantitative response of worms with respect to dopamine specific functions as lack of dopamine severely impairs the chemotactic response of the worms in presence of nonanol. Briefly, when a drop of nonanol, was placed near the head of a normal worm, the worm senses it and moves away [21] **.** However, with the altered dopamine level as in disease condition of PD, the repulsion response time is increased because of altered sensing behaviour. In the present study, the assay was carried out in triplicates for each of the strains and the response time (in seconds) was monitored and the statistical significance was calculated.

### 7. Studies on expression of dopaminergic transporter

*C. elegans dat-1* encodes dopaminergic transporter hence we endeavoured to study the effect of *ida-1* silencing on its expression employing transgenic *C. elegans* strain BZ555 that expresses GFP under the effect of *dat-1* specifically in the dopaminergic neurons [21]. The *ida-1* knockdown adult worms were washed with M-9 buffer, then immobilized using 100 mM sodium azide (Sigma, Cat no. 71289) and mounted on agar padded glass slides. Fluorescent imaging for GFP was carried out using fluorescent microscope (Carl Zeiss).

### 8. Assay on locomotor activity

The effect on locomotion via neurotransmitter imbalances, is a hallmark of PD, hence we employed thrashing assay to quantify the motility of worms. In this assay the number of thrashes of worms was counted for a specified time; a single thrash was defined as the bending of the body to the outermost angle and then back to the initial posture. After 48 h incubation, harvested adult animals were washed with M-9 buffer. Then a small volume of this worm suspension was transferred on to the glass slide; 10 worms were randomly selected, allowed to be stabilised in M-9 buffer for 30 seconds and each of the worms was analysed singly for its motility in a time-period of 30 seconds [22]. The mean number of thrashes ± SE for each group was calculated and the observations were evaluated statistically and represented graphically.

### 9. Estimation of oxidative stress

Reactive oxygen species (ROS) formation was quantified using 2,7- dichlorodihydrofluorescein-diacetate (H_2_-DCF-DA) [23] in the control vs. *ida-1* knockdown worms. Approximately 100 worms in about 40–60 µL phosphate buffered saline were transferred to assay well of 96 well plate, followed by making up of the volume to100 µL and addition of 100 µL 2,7- dichlorodihydrofluorescein diacetate (H_2_DCFDA)(Invitrogen, Cat. No. D399) so as to achieve a final concentration of 50 µM DCFDA. Fluorescence was read initially without the dye, at the time of adding the dye and one hour after addition of dye, using fluorimeter {485 excitation, 520 emission}. Initial readings were subtracted from the final readings and fluorescence per 100 worms was calculated.

### 10. Assay for DAF-16 nuclear localization

The quantification of DAF-16 nuclear localization, was suitably studied in transgenic TJ-356 animals (DAF-16::GFP), after raising them on control and ida-1 treated RNAi plate. For heat shock experiment, the worms were incubated at 35°C for 30 minutes [24]. GFP quantification was carried out at 40X magnification by fluorescence microscope (Carl Zeiss, Axio Vision M2); translocation of DAF-16 was scored by assaying the presence of GFP accumulation in the nuclei.

### 11. Quantitative Real Time PCR (qPCR) studies for *daf-2* and *daf-16* mRNA expression

The total RNA of the worms from control and experimental groups was extracted followed by preparation of cDNA and quantification of mRNA levels of daf-2 and daf-16, employing qPCR. [25]. In brief, for RNA preparation, harvested worms were washed with DEPC (Sigma, Cat. No.D5758) treated water, and the RNA was isolated using RNAzol (Molecular Research Center, Cat. No.RN190) reagent. Isolated total RNA was then subjected to cDNA synthesis using Revert Aid Premium First Strand cDNA Synthesis Kit (Fermentas, Cat. No. K1652). The cDNA was used at a concentration of 125 ng and processed for quantitative-real time PCR using Sybr green master mix to quantitate the expression of *daf-2* and *daf-16*, using actin as the loading control. The primers used were.

*act-1*: Forward TTACTCTTTCACCACCACCGCTGA.

Reverse TCGTTTCCGACGGTGATGACTTGT.

*daf-2*: Forward TTCAACACGGAATCAGGGTGTCCT.

Reverse AGACGAGAAGCATGCCGAGAATGA.

*daf-16*: Forward GCGAATCGGTTCCAGCAATTCCAA.

Reverse ACACGATCCACGGACACTGTTCAA.

The amplification in 96-well plate was done with the programme as: (1) 1 cycle of Pre-incubation: 50°C for 2 minutes and 95°C for 10 minutes; (2) 40 cycles for Amplification: 95°C for 30 seconds, 55°C for 30 seconds, 72°C for 30 seconds; (3) Melting curve analysis: 95°C for 5 seconds, 65°C for 1 minute, (4) Cooling: 40°C for 5 minutes. After amplification, the Ct values were obtained which were used for the relative quantification of the expression of the target genes. The relative quantification was based on 2^−ΔΔCt^ method.

### 12. Statistical analysis

The graphical data were presented as mean ± standard error of the mean. Data with multiple groups i.e. α-syn aggregation under various treatment were analysed using one way ANOVA followed by Tukey test which compares every mean with every other means of the groups. The data between two groups were statistically analysed employing Student’s t-test by Graph Pad Prism 5 software.

## Results

### 1. Silencing of *ida-1* led to significantly increased α-syn aggregation in the transgenic *C. elegans* strain

**Figure 1:**
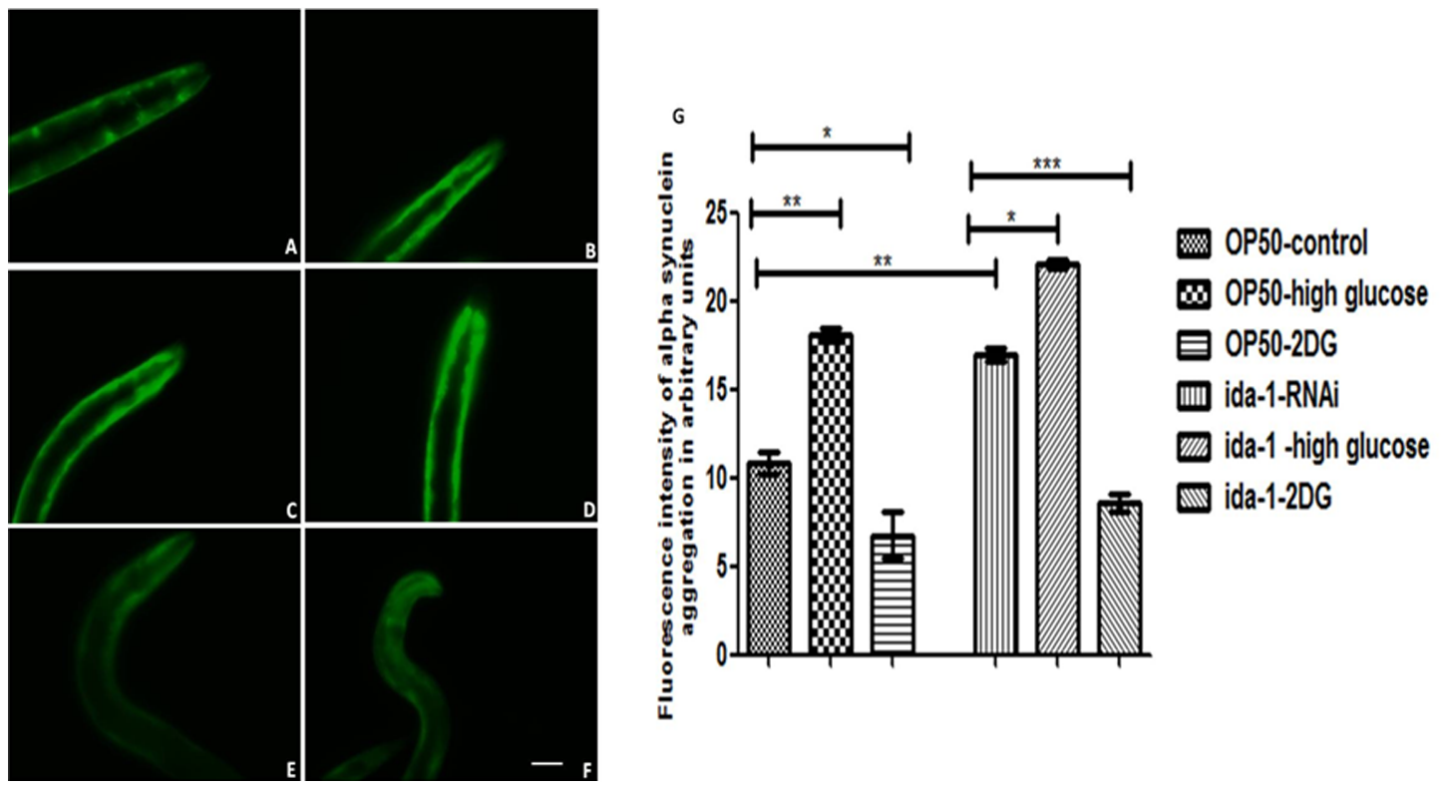
α-syn aggregation in NL5901 strain of *C. elegans*. Worms treated with OP50 (A); *ida*-1 knockdown (B); control- 14 mM/L Glucose (C); *ida*-1 knockdown −14mM/L Glucose (D); control worms raised on 50 mM DOG (E); and *ida*-1 knockdown worms raised on 50 mM DOG (F). Figure 1(G) is the graphical representation of mean fluorescence intensity for α-syn aggregation of the nematodes as quantified using Image J software. *p<0.05, **p<0.01, ***p<0.001. Scale bar, 50 µm.

RNAi mediated silencing of *ida-1* led to increase in α-syn aggregation measured as the expression pattern of YFP in the transgenic strain NL5901. Figure 1A is the representative image for YFP expression in transgenic worms raised on OP50 diet (control) while Figure 1B shows worms raised on bacteria inducing *ida-1* silencing. The silencing of *ida-1* increased the fluorescent intensity of α-syn aggregation when compared to OP50 fed worms; as evident by 1.57 fold (P<0.01) increase in the aggregated protein since the mean fluorescence intensity for the control group was 10.81 arbitrary units and that for *ida-1* knockdown worms was 16.98 arbitrary units. When higher glucose was made available to the worms, it resulted in increase in α-syn aggregation for in OP50 fed and as well as *ida-1* knockdown conditions as indicated by Figure 1C & D, respectively. For the control worms, as mentioned earlier, the mean fluorescence intensity was 10.81 arbitrary units, which increased further to 18.12 when glucose was supplemented to the medium; thus showing 1.67 fold increase (P<0.01) in the aggregation pattern. Similarly for the *ida-1* knockdown worms co-supplemented with glucose exhibited an increase in the aggregation level as compared to *ida-1* silencing alone. The *ida-1* knockdown worms exhibited 16.98 arbitrary units of mean fluorescence intensity, which rose to 22.05 arbitrary units for *ida-1* silencing coupled with glucose supplement. When the glucose condition was restricted, the protein aggregation lowered, as observed in Figure 1E (OP50 along with 2 DOG) and Figure 1F (ida-1 RNAi along with 2-DOG). Also the reduction in aggregation intensity was significant for the OP50 worms, giving 6.743 arbitrary units of mean fluorescent intensity On the contrary, in *ida-1* RNAi together with restricted glucose condition mean fluorescent intensity decreased to 8.601 arbitrary units as against 16.98. Thus, it becomes evident that restricting glucose concentration has profound effect on decreasing aggregation intensity both in control OP50 and RNAi treatment, giving 1.6 and 1.97 fold (P<0.05 and P<0.001, respectively) decrease under limited glucose.

### 2. Effect of *ida-1* silencing on dopamine content

**Figure 2:**
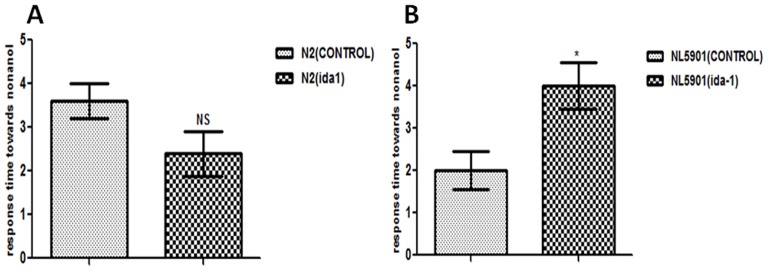
Estimation of dopamine content by employing nonanol assay. *C. elegans* wild type strain N2 {control and *ida*-1 knockdown}(A); Trangenic strain NL5901 expressing human α-syn {control and *ida*-1 knockdown}(B); BL5752 {control and *ida*-1 knockdown}(C); N2 {control} and VC226 {IDA-1 knockout}(D). *p<0.05 and NS - Non significant.

*C. elegans* locomotion is due to coordinated dorsal-ventral muscle contractions that allow the propelling of the nematode in a sinusoidal manner within its environment [26]. *C. elegans* senses temperature, salts, soluble and volatile chemicals, oxygen, mechanical stimuli and other environmental factors that modulate its motion [27], [28]. The dopamine content remained unaltered in the Bristol wild type N2 strain of *C. elegans;* however, in the disease model of *C. elegans* NL5901, dopamine level altered significantly (p<0.05) upon *ida-1* silencing, which was evident by change in the response time of the worm toward nonanol; as represented by Figure 2A and Figure 2B, respectively. The mean ± SEM of the transgenic strain NL5901 was 2.000±0.4472 and that for *ida-1* knockdown NL5901, it was observed to be 4.000±0.5477; indicating a 2-fold increase in the time taken by the *ida-1* knockdown worms to respond towards nonanol.

### 3. Dopamine transporter is not affected by silencing *ida-1*

**Figure 3:**
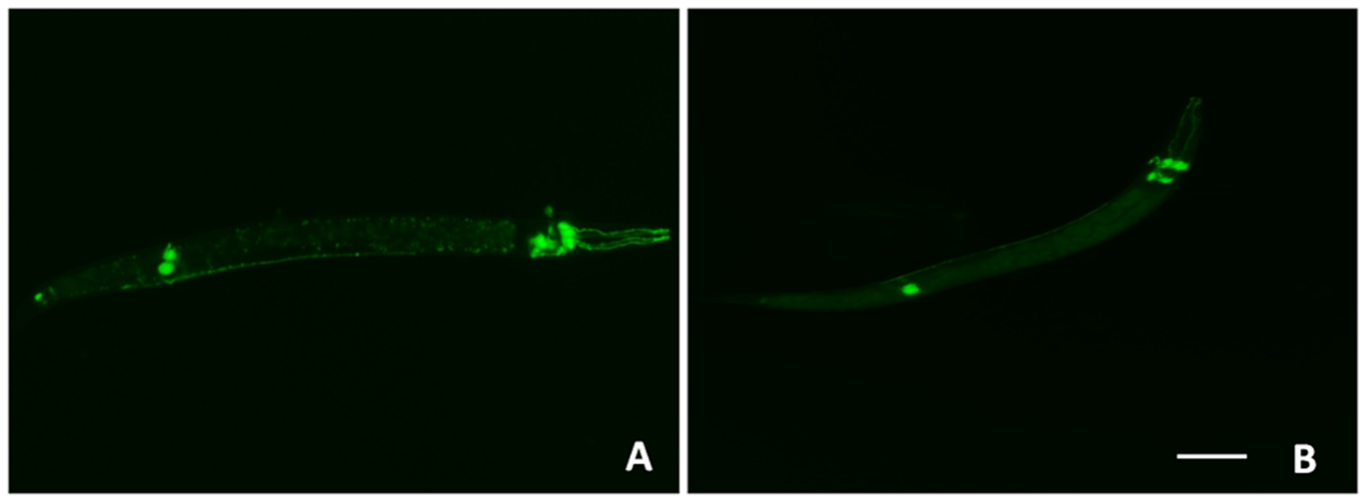
Study for the expression of dopamine transporter (DAT-1). *C. elegans* transgenic strain BZ555 expressing *dat-1* tagged with GFP in control (A) and *ida-1* knockdown worms (B). Scale bar, 100 µm.

The effect of *ida-1* silencing on dopamine transporters was evaluated in the transgenic strain BZ555 but we observed a similar DAT-1::GFP intensity in case of control represented by Figure 3A and the *ida-1* knockdown condition represented by Figure 3B; thus making us believe that dopamine transporter is not affected by *ida-1* silencing.

### 4. Locomotion is significantly reduced upon *ida-1* silencing in NL5901 and in *ida-1* mutant strain

**Figure 4:**
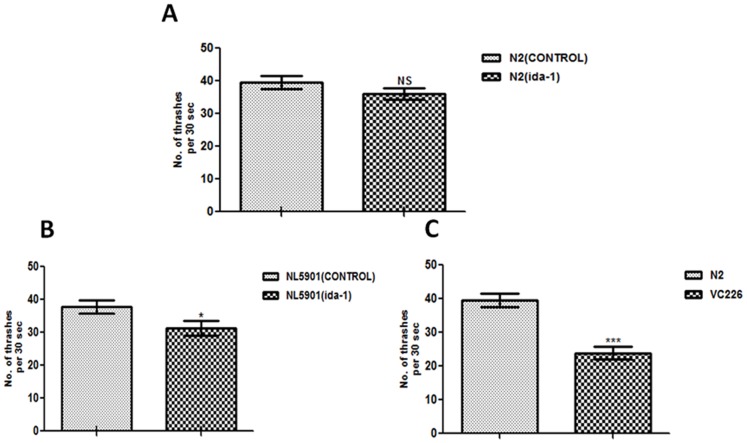
Study for motility as estimated by thrashing assay. *C. elegans* wild type worms N2 (A), Trangenic strain NL5901 expressing human α-syn (B), and ida-1 knockout strain VC226 (C). *p<0.05, ***p<0.001, NS - Non significant.

*ida-1* silencing showed no marked changes in the number of body movements in the N2 wild type isolate as represented by Figure 4A. However, the motility significantly decreased in *ida-1* knockdown NL5901 and *ida-1* knockout strain VC226 as compared to the OP50 fed NL5901 and N2, respectively. The result obtained upon performing student’s t-test (p<0.05) indicated the slight reduction of 1.2 fold in the number of thrashes for transgenic NL5901 with mean ± SEM for control 37.80±1.931and 31.30±2.246 for the *ida-1* knockdown group as indicated by Figure 4B. The number of thrashes reduced drastically in the Ida-1 knockout strain VC226, thrashing count of 24 per 30 seconds (p<0.001) was observed against 43 thrashes in the control N2 group for the same duration, represented graphically by Figure 4C.

### 5. ROS levels are not altered by silencing of *ida-1*

**Figure 5:**
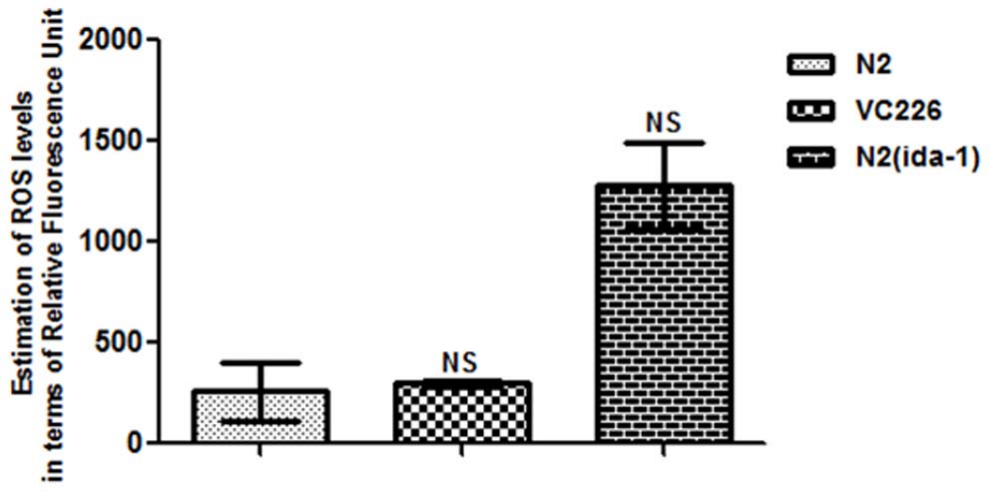
ROS production levels as estimated by H_2_-DCF-DA assay. *C. elegans ida-1* knockout strain VC226 and in wild type strain N2 under *ida-1* knockdown condition. NS: Non-significant.

Though, *ida-1* silencing resulted in increased aggregation of α-syn, it did not affect the formation of reactive oxygen species (ROS) within the worms as represented graphically with Figure 5. The results clearly indicate the non-significant levels of ROS as estimated by relative fluorescence units normalized with respect to control, both in case of *ida-1* knockdown N2 worms and also the *ida-1* knockout VC226 strain of *C. elegans*. The mean ± SEM of control N2 was 257.1±142.9 and that for *ida-1* knockdown N2 and VC226 mean ± SEM was 297.9±12.50 and 297.9±12.50, respectively.

### 6. *ida-1* silencing caused markedly reduced Daf-16 nuclear localization

**Figure 6:**
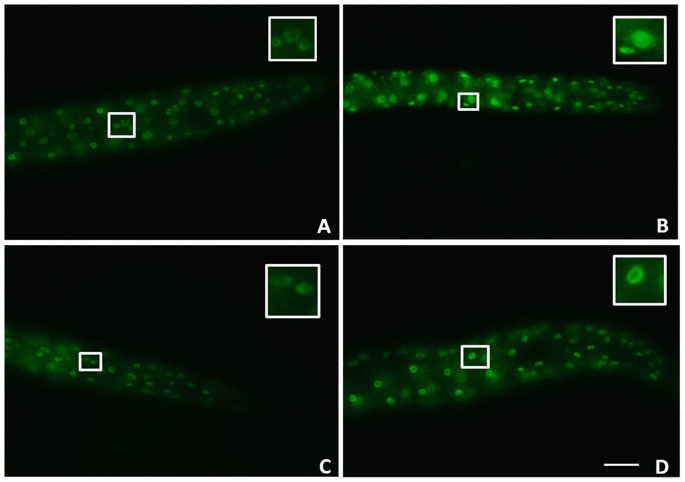
DAF-16 nuclear localization in *C. elegans* transgenic strain TJ-356 expressing DAF-16::GFP. Worms fed on OP50 (A); Worms fed on OP50 and subjected to heat shock (B); *ida-1* knockdown worms (C) worms grown on *ida-1* RNAi bacteria and subjected to heat-shock (D). Scale bar, 50 µm.

The translocation of DAF-16 forkhead transcription factor from cytoplasm to the nucleus, is negatively regulated by its upstream effector DAF-2; and the cytoplasmic retention of DAF-16 is a major determinant of life-span. GFP intensity for DAF-16 expression gets sufficiently reduced in the case of *ida-1* knockdown adult animals as compared to the worms raised on *E. Coli* OP50 diet; as indicated by the representative images for control (Figure 6A&B) and *ida-1* treatment (Figure 6C&D). The reduction in DAF-16 expression is also supported by the qPCR studies carried out for the mRNA expression of *daf-2* and *daf-16*. Thus, confirmatory logical deductions can be made that upon *ida-1* silencing the DAF-16 levels are significantly lowered, its mechanism might be understood through real-time PCR studies.

### 7. mRNA levels of *daf-2* and *daf-16* are modulated in *ida-1* knockdown worms

**Figure 7:**
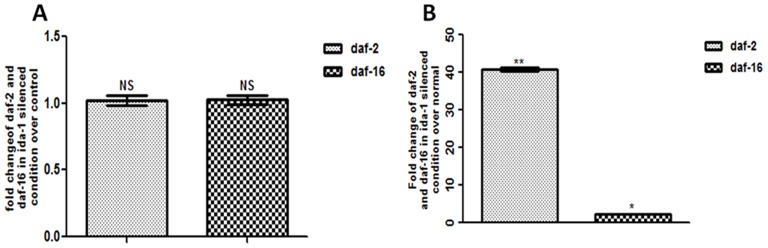
Real time PCR studies to quantify *daf-2* and *daf-16* mRNA levels after *ida-*1 silencing. *C. elegans* wild type strain N2 (A) and trangenic strain NL5901 expressing human α-syn. *p<0.05 **p<0.01, NS - Non significant.

In case of disease model of PD, the transgenic *C. elegans* strain NL5901, results indicate significantly increased expression of *daf-2* and *daf-16* levels. Figure 7A is the graphical representation of *daf-2* and *daf-16* expression level in wild type Bristol N2. Figure-7B shows that upon *ida-1* silencing in PD model of worm NL5901, *daf-2* expression increased approximately 40 folds while the *daf-16* expression showed a 2 fold increase.

## Discussion

We carried out a detailed work-up on the function of *ida-1* vis-à-vis PD and diabetes associated endpoints employing model system *C. elegans*. Our data suggest that Ida-1 has a strong association with the various parameters implicated in PD which are further worsened under increased glucose stress. Our studies with transgenic *C. elegans* strain expressing α-syn revealed an increased aggregation of this PD hallmark protein under *ida-1* knockdown condition. IDA-1, being a Type 1 Diabetes auto antigen, these initial findings made it intriguing for us to further evaluate any potential role of Ida-1 under condition of dysregulated glucose concentration and to assess any potential relation between α-syn aggregation level and diabetes; employing high and restricted glucose conditions for the growth of worms. Moreover, it has been hypothesized that insulin dysregulation contributes to neurodegenerative disorders including PD [29], which results in altered blood glucose level; although the exact mechanism is still unclear. Our result thus establishes a potential protective role of gene *ida-1* in preventing the α-syn aggregation, possibly via functioning of Ida-1 within the machinery that mediates chaperonic activity and reduces the occurrence of misfolded protein aggregates. The results further convey that under the action of high glucose concentration, as in the case of diabetes wherein glucose metabolism is altered, the process of misfolded protein formation is enhanced and thus increased α-syn aggregation in case of PD is significantly evident. These findings provide interesting initial clues leading us to believe that *ida-1* silencing in itself increases the α-syn aggregation. This aggregation pattern seems to be directly linked with glucose concentration since aggregates are manifested under higher glucose concentration and decreased under restricted glucose concentration. These findings provide inferences about lowered observance of aggregation when glucose concentration is minimized which is consistent with previous findings that calorie restriction minimises neuronal cell death [18]. These findings pave way for studying ILS pathway in association with the genes that are affected by *ida-1* and those which are further responsible for protection in neurodegenerative disorders. Considering, α-syn aggregation as a major hallmark of PD, which involves dopamine imbalance; thus we next studied the effect of *ida-1* silencing on dopamine content using a nonanol based repulsion assay. Our findings indicate that the dopamine levels get altered in the PD model of *C. elegans* and upon *ida-1* silencing, worms exhibited the phenotype reflecting reduced dopamine activity. Taking the studies further, we next explored the effect of *ida-1* silencing on dopaminergic transporter by studying the expression of *C. elegans* dopamine transporter DAT-1 which led us to conclude that despite the significant role of *ida-1* in ILS and neurotransmitter release [10], [12], it perhaps might not play any role in affecting the dopamine transporter in the animal model of *C. elegans* as DAT-1 involved in the regulation of dopaminergic neurotransmission via reuptake of dopamine into presynaptic neurons and it is not necessary that DAT must be the only target for reducing dopamine level. The dopamine levels may also be possibly affected by other mechanism. In order to understand the effect of *ida-1* silencing on locomotion via neurotransmitter imbalances, which is a hallmark of PD, we employed thrashing assay to quantify the motility of worms and our findings show decreased motility. This reduction in the number of thrashes can be attributable to the role of Ida-1 in excitatory neurotransmission. When *ida-1* is knockdown, its functional activity is appreciably lost and thus possibly there might be lesser synthesis or release of excitatory neurotransmitters which are strongly associated with the movement and other sensory pathways involved in the nematode *C. elegans* [12]. Next in our study, ROS levels were studied under *ida-1* knockdown condition and our findings infer that amongst the multiple factors associated with PD, Ida-1 might not be involved in the pathways relating to oxidative stress in the context of α-syn expressing *C. elegans* strain.

In *C. elegans*, ILS system is the major pathway involved in the regulation of nematode reproductive life cycle, development, glucose metabolism, longevity, fat metabolism, learning, and stress resistance [30]. In ILS pathway DAF-2 functions as receptor of the ligand while DAF-16 as the downstream target of evolutionary conserved and well-characterized ILS pathway. *daf-2* and *daf-16* encodes the *C. elegans* ortholog of the mammalian insulin/IGF receptor and forkhead box O (FOXO) homologue respectively. The translocation of DAF-16 forkhead transcription factor from cytoplasm to the nucleus is negatively regulated by its upstream effector DAF-2. Considering increased α-syn aggregation upon *ida-1* silencing and taking into account the significance of mammalian genes *IA-2* and phogrin as type-1 diabetes autoantigen, we carried out DAF-16 nuclear localization assay in order to enumerate any potential relationship between Ida-1 and chaperonic/stress resistance activity of DAF-16. DAF-16 is widely reported to play the key role in stress responses and act as potent modulator of α-syn toxicity induced by aggregation in *C. elegans* [31], [32]. In transgenic *C. elegans* strain for Alzheimer’s disease modelwhich eexpresses human amyloid beta, nucleus translocation of DAF-16 has significantly decreased the age-onset aggregation-associated toxicity [33]. Thus upregulation of *daf-16* increases the youthfulness of nematode, maintains protein homeostatsis of misfolded/normal proteins and prevent proteotoxicity induced by protein aggregates and vice versa [14]. In our results we observed that *ida-1* RNAi nematode displayed impaired stress induced DAF-16 nuclear localization. The translocation of DAF-16 to the nucleus was suppressed/inhibited in the absence of *ida-1* in response to heat shock, decreasing the synthesis of stress proteins which are required to confer stress-resistance. By this we conclude that IDA-1 prevents DAF-16 from entering into the nucleus and thus, inhibiting it to promote or repress any transcription of genes required for DAF-2 dependent functions. Furthermore, the interaction between *daf-2* and *ida-1* [10], [34] also explains the neuroprotective effects of IDA-1 via DAF-16 signalling pathway. To fully explore the mechanistic basis of DAF-16 regulation, real-time PCR studies were carried out to assess the mRNA levels of *daf-2* and *daf-16*. The real time PCR studies in transgenic strain NL5901, clearly indicates that *daf-2* expression increases magnificently but only a marginal increase in *daf-16* expression is observed; which when implicated with the ILS pathway, provide strong evidence to link together the reduced levels of *daf-16* and thus the hindered protective effect in enabling proteostasis at the normal rate [35]. DAF-16 is known to be a downstream effector of DAF-2 in ILS pathway, and an increased DAF-16 expression exerts protective effect via enabling proteostasis and inhibiting toxic protein aggregation. However, *ida-1* silencing results in sufficiently high levels of *daf-2*, consequently causing the misfolded proteins to aggregate**,** a critical feature for the occurrence of neurodegenerative disorders [36], which might be the reason for increased α-syn expression. Our studies thus provide evidence for the association of *ida-1* with neurodegenerative PD and that of its potential role in worsening PD associated effects under increased glucose concentrations, thus providing interesting clues towards *ida-1* being a common modulator between PD and diabetes via the DAF-2/DAF-16 ILS pathway.
